# Poliovirus Excretion in Children with Primary Immunodeficiency Disorders, India

**DOI:** 10.3201/eid2310.170724

**Published:** 2017-10

**Authors:** Madhu Chhanda Mohanty, Manisha Rajan Madkaikar, Mukesh Desai, Prasad Taur, Uma Prajwal Nalavade, Deepa Kailash Sharma, Maya Gupta, Aparna Dalvi, Snehal Shabrish, Manasi Kulkarni, Jahnavi Aluri, Jagadish Mohanrao Deshpande

**Affiliations:** Enterovirus Research Centre, Mumbai, Maharashtra, India (M.C. Mohanty, U.P. Nalavade, D.K. Sharma, J.M. Deshpande);; National Institute of Immunohaematology, Mumbai (M.R. Madkaikar, M. Gupta, A. Dalvi, S. Shabrish, M. Kulkarni, J. Aluri);; Bai Jerbai Wadia Children’s Hospital, Mumbai (M. Desai, P. Taur)

**Keywords:** enterovirus, poliovirus, primary immunodeficiency disorder, oral polio vaccine, OPV, vaccine-derived polioviruses, VDPV, viruses, Mumbai, India, children

## Abstract

Prolonged excretion of poliovirus can occur in immunodeficient patients who receive oral polio vaccine, which may lead to propagation of highly divergent vaccine-derived polioviruses (VDPVs), posing a concern for global polio eradication. This study aimed to estimate the proportion of primary immunodeficient children with enterovirus infection and to identify the long-term polio/nonpolio enterovirus excreters in a tertiary care unit in Mumbai, India. During September 2014–April 2017, 151 patients received diagnoses of primary immunodeficiency (PID). We isolated 8 enteroviruses (3 polioviruses and 5 nonpolio enteroviruses) in cell culture of 105 fecal samples collected from 42 patients. Only 1 patient with severe combined immunodeficiency was identified as a long-term VDPV3 excreter (for 2 years after identification of infection). Our results show that the risk of enterovirus excretion among children in India with PID is low; however, systematic screening is necessary to identify long-term poliovirus excreters until the use of oral polio vaccine is stopped.

Oral polio vaccine (OPV) has been a key factor in global polio eradication and has proven highly effective because of its ease of administration and ability to transmit to secondary contacts (*1**–**4*)*.* Primary immunodeficiencies (PIDs) are a heterogeneous group of inherited disorders resulting from developmental defects or dysfunction of the immune system components (*5*). Patients with PID can potentially be infected by immunizations if they receive live vaccines (*6*). OPV immunization has been associated with poliovirus infection in patients with primary antibody deficiencies and combined immunodeficiencies, which can lead to paralysis (*7**,**8*)*.* In addition, some vaccinated patients with PID may shed vaccine-derived polioviruses (VDPVs) because of a prolonged period of intestinal replication. VDPVs show increased nucleotide divergence in the viral protein 1 (VP1) coding region in all variants of OPV serotypes (PV1, PV2, and PV3); this divergence is associated with increased neuropathogenicity (*9*)*.* More than 100 immunodeficiency-related VDPV (iVDPV) infections have been reported in PID patients worldwide to date (*10**,**11*). As potential reservoirs for neurovirulent VDPV strains, patients with PID represent a global risk to nonimmunized contacts and to the Global Polio Eradication Initiative (*10**,**12*)*.*

Several studies have investigated iVDPVs in patients with different types of immunodeficiencies, such as combined immunodeficiency, antibody deficiency, and other immunodeficiencies (*8**,**10**,**13**,**14*). Recognizing the risk that iVDPV poses to global poliovirus eradication, the World Health Organization (WHO) maintains a registry of known iVDPV cases and promotes global surveillance of iVDPVs (*15*)*.* However, it is difficult to estimate the number of iVDPV excreters worldwide in the absence of systematic screening of immunodeficient patients for enterovirus excretion, especially in the developing world (*15*)*.*

To establish screening facilities for children with PID in India, we collaborated with the National Institute of Immunohaematology (NIIH, the referral laboratory for PID in India) and Bai Jerbai Wadia Children’s Hospital (a tertiary care unit) in Mumbai, India, which has established facilities for diagnosis of PIDs. The study aimed to identify the proportion of children with PID who have enterovirus infection and also to identify long-term polio/nonpolio enterovirus excreters among them.

## Materials and Methods

### Study Design

The study is being conducted in collaboration with the clinicians of the PID surveillance group at NIIH and Wadia Children’s Hospital. This article describes the data regarding children with PID who were enrolled in the study during September 2014–April 2017. Blood samples were collected at Wadia Children’s Hospital from patients (<18 years of age) suspected of having immunodeficiencies, as part of routine surveillance; diagnosis was confirmed by standard procedure at NIIH.

We obtained ethical clearance from the Ethics Committee of NIIH, Wadia Children’s Hospital, and Enterovirus Research Centre (ERC), Mumbai, and obtained informed consent from the patients’ parents. The investigations were guided by the clinical presentation, immunological abnormalities, and molecular diagnosis wherever feasible, according to the phenotypic classification of the International Union of Immunological Societies (affiliated with WHO) (*16*). Initial investigations involved a complete blood count with a differential count on the leukocytes and mean platelet volume on a Sysmex XS-800i 5-part automated hematological analyzer (Sysmex Co., Kobe, Japan); serum immunoglobulin estimation (IgG, IgA, IgM, IgE) by nephelometry (BNProspec, Siemens); nitroblue tetrazolium blood test by microscopy; and lymphocyte subset analysis by flow cytometry–using BD Multitest 6-color TBNK reagent (BD Biosciences, San Jose, CA, USA) to determine percentages and absolute number of B cells (CD19); T cells (CD3); T-helper cells (Th, CD3, and CD4); T-cytotoxic cells (Tc, CD3, and CD8); and natural killer (NK) cells (CD3 negative and CD16 or CD56). We performed flow cytometry–based assays on FACS ARIA-I using the stain–lyse–wash method and analyzed them with FACS Diva software (BD Biosciences). We made specific PID diagnoses on the basis of clinical features and laboratory investigations (*16**,**17*)*.*

We collected fecal samples from the children with PID who routinely visit the PID outpatient department of Wadia Children’s Hospital for checkup and intravenous immunoglobulin (IVIG) treatment. We followed up with these patients regularly for monthly fecal samples (1 sample in each month for 2 consecutive months); both Wadia Children’s Hospital and ERC staff reminded patients to return with their samples. ERC performed fecal sample processing (as described later), culture, isolation, and characterization of virus isolates. We followed up by taking monthly samples from the patients whose samples were found positive for enteroviruses until 2 consecutive samples became negative for enteroviruses. We followed up again with those patients for enterovirus detection after a 6-month interval.

### Enterovirus Isolation

We performed enterovirus isolation from fecal extracts as described in the WHO laboratory manual (*18*)*.* We used human rhabdomyosarcoma (RD) and transgenic mouse cell line expressing polio receptor (L20B) for enterovirus culture. We treated fecal extracts (10%, wt/vol) prepared in phosphate-buffered saline (Sigma cat. no. D8662; Sigma-Aldrich, St. Louis, MO, USA) with 10% (vol/vol) chloroform before inoculation in cell cultures. We infected cells in duplicate with 200 μL of stool extract, incubated at 36°C, and observed microscopically for cytopathogenic effect (CPE) daily for 5 days. We freeze-thawed cell cultures showing CPE and collected culture medium for virus identification. We scored samples as negative if 2 consecutive passages in the same cell line did not produce CPE.

### Enterovirus Typing

We extracted viral RNA from culture medium of CPE-positive freeze-thawed cells using a QIAamp Viral RNA Mini Kit (QIAGEN, Hilden, Germany) according to the manufacturer’s instructions. For nonpolio enteroviruses, we used primer pairs 222/224 and 88/89 for partial VP1 sequencing for identification of enterovirus type (*19**,**20*). For poliovirus isolate VP1 region amplification (≈900 nt), we performed reverse transcription PCR in a single tube using reverse primer Q8 and forward primer Y7, as described previously (*21*). We performed the sequencing using a BigDye Terminator v3.1 Cycle Sequencing Kit (Applied Biosystems, Foster City, CA, USA) according to the manufacturer’s instructions. We resolved the sequences on an ABI 3130xl Genetic Analyzer (Applied Biosystems) and edited them using Sequencher version 4.10.1 software (Gene Codes, Ann Arbor, MI, USA). For nonpolio enteroviruses, we subjected the sequences to BLAST search (https://www.ncbi.nlm.nih.gov/BLAST) and defined the virus type using the criteria of >75% nucleotide and >85% amino acid similarity in the VP1 region (*22*). For poliovirus isolates, we compared sequences with the Sabin reference strain in the VP1 region.

## Results

A total of 1,393 patients with suspected PID were screened at the PID Outpatient Department of Wadia Children’s Hospital during a period of 2 years and 7 months; of these, the NIIH PID screening group confirmed that 151 patients had PID. We were able to follow up with 42 patients with humoral, combined, and other PIDs for fecal sample collection to test for enterovirus excretion. There were 33 male and 9 female participants. We found a striking predominance of male participants in all types of PID except IgG subclass deficiency. The age of the study participants ranged from 4 months to 18 years of age (Table 1). The PID types showed a mixture of antibody deficiencies, combined T and B cell deficiencies, phagocytic defects, and other immunodeficiencies (Table 1).

**Table 1:** Baseline characteristics of confirmed PID cases in children recruited from Wadia Children’s Hospital, Mumbai, India, September 2014–April 2017*

Serial no.	PID types	No. cases	M/F ratio	Age range, mo
1	Hemophagocytic lymphohistiocytosis	11	10/1	4–96
2	X-linked agammaglobulinemia	7	7/0	27–216
3	Chronic granulomatous disease	4	3/1	7–60
4	Severe combined immunodeficiency	4	4/0	4–48
5	Common variable immunodeficiency	3	2/1	84–120
6	Chédiak–Higashi syndrome	3	1/2	26–86
7	Hypogammaglobulinemia	2	1/1	96–108
8	IgG subclass deficiency	2	0/2	84–114
9	Hyper-IgM syndrome	2	2/0	17–59
10	Autoimmune lymphoproliferative syndrome	1	0/1	15
11	B cell expansion with NF-κB and T cell anergy	1	1/0	18
12	Hyper-IgE syndrome	1	1/0	42
13	Interleukin 12 receptor β1 defect	1	1/0	156
**Total**		42	33/9	4–216

*PID, primary immunodeficiency

Of 42 children with PID, 40 (95%) had received OPV 1–96 months before fecal specimen collection; the parents of the remaining 2 (5%) children were not able to recall whether their children had received OPV. All patients with antibody-mediated immunodeficiency were under replacement IVIG prophylaxis. We collected fecal samples before the monthly IVIG therapy was administered. Of the 42 patients enrolled for the study, 5 (11.9%) underwent bone marrow transplant and 10 (23.8%) died during the study period.

We received 105 fecal samples from 42 patients with confirmed PID; we tested these for polio and other enteroviruses. Among the fecal samples from these 42 patients, specimens of 8 patients (19%) tested positive for enteroviruses. Of these, we found 1 patient with severe combined immunodeficiency (SCID) who had excreted poliovirus for 2 years and then abruptly stopped excreting it. Fecal specimens of a 4-month-old patient with SCID and 1 patient with familial hemophagocytic lymphohistiocytosis (FLH) tested positive for poliovirus (Sabin type 1) (Table 2). A total of 4 patients with SCID were enrolled in this study; SCID was diagnosed in 2 of them at 2 months of age, and fecal specimens collected for detection of enterovirus excretion were found negative. Both the patients died before the second sample collection. Samples from 3 patients with antibody deficiencies and 2 patients with other immunodeficiencies tested positive for nonpolio enterovirus (NPEV), but subsequent samples tested negative (Table 3). None of the samples from patients with common variable immune deficiency (CVID) tested positive for enterovirus excretion.

**Table 2:** Demographic and clinical data for patients with PID whose fecal samples tested positive for polioviruses, Wadia Children’s Hospital, Mumbai, India, September 2014–April 2017*

Serial no.	Age, mo/ sex	PID type	Months last OPV†	IVIG therapy	BMT	Results by collection day
1	48/M	SCID	37	Yes	ND	D1, VDPV3; D91, VDPV3; D175, VDPV3; D207, VDPV3; D263, VDPV3; D334, VDPV3; D369, VDPV3; D454, VDPV3; D488, VDPV3; D524, VDPV3; D550, VDPV3; D634, VDPV3; D700, VDPV3; D774, neg; D799, Neg; D840, neg; D930, neg
2	48/M	FLH	NA	No	ND	D1, neg; D49, P1SL; D128, neg; D241, neg
3	4/M	SCID	4	Yes	ND	D1, P1SL; D29, P1SL

*BMT, bone marrow transplant; D, day of collection following first collection; FLH, familial lymphohistiocytosis; IVIG, intravenous immunoglobulin; NA, not available; ND, not done; neg, negative; OPV, oral polio vaccine; P1SL, polio1 Sabin-like; P3SL, polio 3 Sabin-like; PID, primary immunodeficiency disease; SCID, severe combined immunodeficiency; VDPV3, type 3 vaccine-derived poliovirus.  †Time from last OPV to first fecal sample collection.

**Table 3:** Demographic and clinical data for patients with PID whose fecal samples tested positive for nonpolio enteroviruses, Wadia Children’s Hospital, Mumbai, India, September 2014–April 2017*

Serial no.	Age, mo/ sex	PID type	Months from last OPV†	IVIG therapy	BMT	Results by collection day
1	114/F	IgG subclass deficiency	60	Yes	ND	D1, neg; D62, EV75; D214,neg; D250, neg; D419, neg; D476, neg; D685, neg
2	42/M	Hyper-IgE syndrome	24	No	ND	D1, E13; lost to follow-up
3	18/M	BENTA disease	NA	No	ND	D1, E5; D185, neg; D273, neg
4	30/M	CGD	NA	No	ND	D1, E14; D10, died
5	54/M	XLA	1	Yes	ND	D1, EV76; D107, neg; D136, neg; D260, neg

*BENTA, B cell expansion with NF-κB and T cell anergy; BMT, bone marrow transplant; CGD, chronic granulomatous disease; D, day of collection following first collection; E5, echovirus 5; E13, echovirus 13; E14, echovirus 14; EV75, enterovirus 75; EV76, enterovirus 76; IVIG, intravenous immunoglobulin; NA, not available; ND, not done; neg, negative; OPV, oral polio vaccine; PID, primary immunodeficiency disease; XLA, X-linked agammaglobulinemia. **†Time from last OPV to first fecal sample collection.**

### Poliovirus Excretors

A 6-year-old boy with a case of leaky SCID had been excreting poliovirus for 2 years. Genetic sequencing of virus isolates identified type 3 VDPV with up to 41 (4%) nt changes at 4 years of age, which subsequently showed up to 93 (10%) nt divergence in VP1 region (from the parental Sabin strain) at 6 years of age. After a prolonged excretion for 2 years, the child abruptly stopped excreting; subsequent collections were found negative for poliovirus. The child had received routine immunizations, as well as Pulse Polio doses on National Immunization Days, until he was 14 months of age; he was also immunized with IPV at 3.5 years of age. The first and second samples from a 4-month-old child with SCID tested positive for Sabin1 poliovirus (separate samples collected each month until patient becomes negative for enterovirus excretion); as of this writing, the third sample is due for collection. The fecal specimen of another patient with FLH tested positive for poliovirus in the second sample, but 2 subsequent samples were negative (Table 2). This child was being treated for FLH but had peripheral demyelinating neuropathy unrelated to this disease, and died 5 days after the fourth specimen collection.

One patient with X-linked agammaglobulinemia (XLA) was enrolled in our study; this patient had a previous record of VDPV2 excretion detected by acute flaccid paralysis (AFP) surveillance by the National Polio Surveillance Program (NPSP, WHO) being conducted at our center. The child had a paralytic attack 3 months after receiving the last dose of OPV, 2 years before enrollment in our study. Case history showed detection of VDPV2 through AFP surveillance, with 7 nt changes from the parent virus. The child was routinely receiving replacement IVIG therapy because of suspected PID; subsequent specimens tested for poliovirus excretion were found negative. No virus shedding was detected in the monthly specimens collected after this child was enrolled in our study.

### Nonpoliovirus Excreters

Five (12%) patients tested positive for nonpolio enteroviruses; 1 patient with IgG subclass deficiency excreted enterovirus 75 (EV-75) and 1 patient with XLA excreted EV-76 in only 1 specimen each. These 2 patients were receiving IVIG replacement therapy and being followed up for enterovirus excretion. A patient with the rare immunodeficiency disorder dedicator of cytokinesis 8 deficiency (DOCK8 deficiency, or hyper IgE syndrome) excreted echovirus 13 in the first fecal sample, and another patient with the rare immunodeficiency disorder B cell expansion with NF-κB and T cell anergy (BENTA) disease excreted echovirus 5 in the first fecal sample. The patient with DOCK8 deficiency was lost to follow-up; repeated monthly samples from the patient with BENTA disease tested negative for enterovirus. The first specimen from a patient having chronic granulomatous disease, a phagocytic disorder, tested positive for echovirus 14, but the patient died before the next specimen collection (Table 3).

## Discussion

Children with PID are at risk for prolonged infection with enteroviruses and may excrete iVDPV after receiving OPV or after being exposed to contacts excreting poliovirus. Such patients are at risk for developing paralytic poliomyelitis and must be identified, as they may pose a risk of reintroduction of the virus into the population after global eradication of poliovirus.

The first objective of this study was to identify chronic or long-term poliovirus/nonpolio enterovirus excreters among children with various PIDs being treated at a tertiary care unit in Mumbai. Although there was no chronic (>5 years) poliovirus excreter among the participants, we identified 1 patient with SCID as a long-term (>6 months) VDPV3 excreter who was excreting VDPV for 2 years. One of the notable highlights of our study was that 6 (75%) of 8 children with PID who were excreting enterovirus had combined or other immunodeficiencies, rather than only antibody-mediated immunodeficiency. Madkaikar et al. reported the distribution pattern of PID in the same tertiary care unit in Mumbai, which varied considerably from those reported by United States, Europe, Africa, and other Asian countries, as follows: diseases of immune dysregulation (29%), phagocytic defects (29%), predominant antibody deficiency (13%), combined T and B cell deficiency (19%), and other well-defined diseases (10%) (*17*). Our study showed a similar pattern of distribution in PID participants: diseases of immune dysregulation (31%), predominant antibody deficiency (34%), phagocytic defects (17%), and combined T and B cell deficiency (14%). Except for 1 patient (a VDPV2 excreter), none of the participants had paralytic disease at the time of enrollment in the study or developed paralysis during the study period.

The VDPV3 isolates excreted by the patient with SCID showed 4% nucleotide change at 4 years of age, indicating that the source of virus was most probably the OPV given at birth. The patient has been receiving IVIG at regular intervals since he received the diagnosis of SCID. In an outbreak of poliovirus infection in Minnesota, USA, in 2005, a patient with PID continued to shed iVDPVs while receiving immunoglobulin therapy for several months and finally stopped shedding virus after a second bone marrow transplant (*23*). According to the WHO Update on VDPVs (*24**,**25*), 6 patients with SCID, 5 of them with no paralytic manifestations, were excreting VDPV2 for a long time (>4 months to several years), and 1 continued virus excretion even after a bone marrow transplant. Unlike a Sri Lanka study (*14*) that reported that children with SCID may not pose a threat to the community because of their short life span, our study, along with studies in Israel, Iran, Libya, and Turkey (*24**,**25*), showed that some patients with SCID can excrete the virus for a prolonged period even after bone marrow transplant, with a high rate of nucleotide changes from the parent Sabin strain. The life expectancy of these children varies depending on the type of SCID/mutation, so it is necessary to analyze the immunological workup of these patients for visualizing whether the child can survive or excrete viruses. Another striking feature common to these SCID poliovirus excreters is that most of them excrete VDPV without exhibiting any paralytic symptoms. Of the 4 participants with SCID in our study, 2 of them received their diagnoses at 2 months of age; their fecal specimens were found negative for enteroviruses, but the infants died before the next sample collection at the age of 4 months. One reason for the survival of 1 child with SCID could be that his condition was diagnosed as leaky SCID (*26*), whereas the 2 infants who died exhibited classical SCID. Further studies on the patient with leaky SCID are in progress to enumerate the changes in immunological parameters that might have contributed to stopping virus excretion. The information about this child was provided to NPSP for screening of contacts and other precautionary measures.

FLH accounted for the largest number of patients in our study, which enrolled 11 patients with FLH. The reason could be that the FLH treatment protocol requires frequent hospitalization and follow-up, so the patients with FLH were therefore accessible for fecal sample collection. One of the children with FLH excreted Sabin1 poliovirus only once, and subsequent specimens tested negative.

The incidence of agammaglobulinemia has been estimated to be ≈1/100,000 live births in the United States and Europe, but no estimates have been made for developing countries (*27*)*.* We enrolled 7 patients with XLA (17%), ranging in age from 20 months to 18 years; all of them had received routine OPV and are currently undergoing IVIG replacement therapy. One patient with XLA had a history of early demyelination with Guillain-Barré syndrome and onset of paralysis; VDPV2 was detected in this patient at 10 months of age through the WHO AFP surveillance program. Aside from patients with SCID, patients with B cell–mediated PIDs such as XLA and CVID have been reported to excrete VDPVs (*14**,**25**,**28*)*.* There are controversies regarding replacement IVIG therapy preventing enterovirus infections in B cell deficiency disorders (*29**,**30*)*.* In our study, the role of IVIG in stopping VDPV shedding cannot be ruled out for the patient with SCID who excreted VDPV3 and the patient with XLA who excreted VDPV2.

Eight (19%) of 42 participants were excreting enteroviruses with no associated disease, which can be considered normal for most enterovirus infections. The excretion of enteroviruses in our study was not restricted to antibody-mediated immunodeficiency; rather, it included patients with different spectrums of PID, although there was only 1 long-term excreter. Three patients had CVID, but none of them has excreted enteroviruses so far, as reported in other studies (*14**,**31*)*.* The reason could be that all 3 participants received their diagnoses after the age of 7 years and would thus have had their diagnoses missed by either AFP or PID surveillance, as all of them are asymptomatic (*31**,**32*)*.*

Our data have limitations in that they represent the PID participants in a tertiary care unit in Mumbai, India, restricting the study population to only Mumbai and parts of Maharashtra state. Patient follow-up, sharing of fecal samples, and collection of specimens at regular intervals were challenging because of lack of awareness and financial burden. Although 151 patients with diagnosed PID were recorded during our study period, only 42 could be followed up. Our study does not allow a precise quantification of the risk for prolonged excretion of poliovirus among patients with PID because there was only 1 patient who excreted for >6 months. Because we used the RD and L20B cell lines for isolation of enteroviruses, we could have missed some enteroviruses that are not cultivable in these cell lines. Furthermore, owing to the lack of affordable treatment and expensive clinical management of infants and children with persistent and chronic infections, many young children probably died from PID before diagnosis. Because our study included only children <18 years of age, persons with CVID who often do not develop signs or symptoms of immune deficiency until they are young adults would have been missed (*33*)*.* Some patients with B cell immunodeficiency could not be followed, so this study may not be an accurate reflection of all patients with B cell immunodeficiency disorders.

Our study demonstrated that the proportion of children who have PID and enterovirus infection in the study area is not high, which may indicate that the risk of chronic excretion of poliovirus among patients with PID in India is low. However, owing to sample size limitations, we recommend future studies expanding the scope and intensity of surveillance activities at additional referral hospitals in other areas of the country. A large-scale multicenter study is needed for the determination of the pattern of excretion of poliovirus in children in India who have received diagnoses of PID.
